# Brain‐clinical signatures for vagus nerve stimulation response

**DOI:** 10.1111/cns.14021

**Published:** 2022-11-22

**Authors:** Zhihao Guo, Jiajie Mo, Chao Zhang, Jianguo Zhang, Wenhan Hu, Kai Zhang

**Affiliations:** ^1^ Department of Neurosurgery Beijing Tiantan Hospital, Capital Medical University Beijing China; ^2^ Department of Neurosurgery Beijing Neurosurgical Institute, Capital Medical University Beijing China; ^3^ Beijing Key Laboratory of Neurostimulation Beijing China

**Keywords:** brain‐clinical signature, drug‐resistant epilepsy, response prediction, vagus nerve stimulation

## Abstract

**Aim:**

Vagus nerve stimulation (VNS) is a valuable treatment for drug‐resistant epilepsy (DRE) without the indication of surgical resection. The clinical heterogeneity of DRE has limited the optimal indication of choice and diagnosis prediction. The study aimed to explore the correlations of brain‐clinical signatures with the clinical phenotype and VNS responsiveness.

**Methods:**

A total of 89 DRE patients, including VNS‐ (*n* = 44) and drug‐treated (*n* = 45) patients, were retrospectively recruited. The brain‐clinical signature consisted of demographic information and brain structural deformations, which were measured using deformation‐based morphometry and presented as Jacobian determinant maps. The efficacy and presurgical differences between these two cohorts were compared. Then, the potential of predicting VNS response using brain‐clinical signature was investigated according to the different prognosis evaluation approaches.

**Results:**

The seizure reduction was higher in the VNS‐treated group (42.50%) as compared to the drug‐treated group (12.09%) (*p* = 0.11). Abnormal imaging representation, showing encephalomalacia (*p*_corrected_ = 0.03), was commonly observed in the VNS‐treated group (*p* = 0.04). In the patients treated with VNS, the mild/subtle brain abnormalities indicated higher seizure frequency (*p* = 0.03) and worse VNS response (*p* = 0.04). The partial least square regression analysis showed a moderate prediction potential of brain‐clinical signature for VNS response (*p* < 0.01). The increase in the pre‐VNS seizure frequency and structural etiology could indicate a worse prognosis (higher McHugh classification).

**Conclusion:**

The brain‐clinical signature illustrated its clinical potential in predicting the VNS response, which might allow clinicians to personalize treatment decisions for DRE patients.

## Graphical abstract

The Brain‐clinical signature has shown promise to predict the VNS response. This study showed mild brain structural alterations are likely to be associated with greater disease severity and worse VNS response as normal MRI might show highly epileptogenic lesions but are hard to detect.

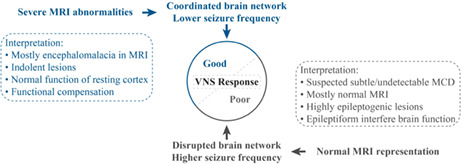

## 1 INTRODUCTION

Epilepsy occurs as a result of abnormal electrical brain activity, causing the malfunctioning of the brain. The epileptic patients have uncontrolled, repetitive seizures that sustain mortality, disability, psychosocial isolation, and a lower quality of life.^1^, ^2^ Antiseizure medications (ASMs) are the first‐line treatment options for epilepsy.^3^ However, approximately one third of epileptic patients suffer from refractory epilepsy, which cannot be controlled by two or more appropriately selected and adequately administered medications and is referred to as drug‐resistant epilepsy (DRE).^4^ Patients with DRE might be benefited from surgical intervention, such as the elimination of seizures after surgical resection or ablation.^5^, ^6^, ^7^ However, for patients, having primary generalized seizures, multifocal or nonlocalizable seizure onset, or epileptogenic zone, involving the eloquent cortex, the surgical resection is contraindicated or ineffective.^8^ Therefore, neuromodulation therapy, such as vagus nerve stimulation (VNS), has emerged as a promising treatment option.^9^ VNS is a palliative treatment, which might alleviate the symptoms of seizures and decrease hospitalizations and psychosocial comorbidities among many epileptic patients. Previous studies reported that VNS could improve the quality of life of the patients, such as their alertness, postictal state, cluster seizures, mood change, verbal communication, school/professional achievements, and memory.^10^ In the past two decades, despite its widespread use with more than 100,000 procedures performed worldwide to date, the type of patients, benefiting from VNS, is still unclear. Patients with DRE show a highly variable and unpredictable response to VNS. A meta‐analysis of 2869 patients across 78 studies demonstrated that approximately 60% of the epileptic patients could achieve a seizure reduction of ≥50% seizure after 2–4 years, while only 8.2% of the patients showed complete elimination of seizures.^11^

In clinical practice, the identification of patients, who might benefit from VNS, is crucial but difficult. This might be due to the different etiologies and clinical phenotypes of the patients who underwent VNS, thereby making the prediction of response to VNS difficult. Determining the patients, who might benefit from VNS, is conducive to reducing unnecessary anesthetic and surgical risks. In addition, this might also exclude those patients, who might not benefit from the treatment, thereby leading to improve the patient's prognosis and treatment level. Therefore, searching for novel biomarkers for the prediction of seizure responsiveness to VNS before surgery is needed for guiding clinical decision making and resource allocation.

Other than clinical parameters, neuroimaging techniques can also provide useful information for VNS surgery and prognostic prediction of epilepsy. A meta‐analysis presented four potential biomarkers of VNS responsiveness, including network/connectomic‐based biomarkers, electrophysiological signatures, structural findings based on neuroimaging, and systemic assays.^12^ However, the patients, who underwent VNS, showed extreme structural abnormalities identified using magnetic resonance imaging (MRI) because a large proportion of the patients suffered from neonatal intracranial hemorrhage and encephalitis. This is the main reason, suggesting the neuroimaging‐based phenotypic identification of VNS or predicting the challenges in VNS response. With the advancements in bioinformatics technologies, researchers can mine more information from neuroimaging‐based images. Specifically, this study could quantify the structural deformations and evaluate brain function statuses, which might be conducive to selecting suitable candidates and supporting the indications, underpinning VNS.

This study cross‐sectionally investigated the demographic characteristics of the patients treated with VNS and pure drugs and explored the brain‐clinical signature of VNS response prediction.

## 2 METHODS

### 2.1 Subjects

This study was reviewed and approved by the institutional review board of Beijing Tiantan Hospital (KY2022‐016‐02). The study was conducted in accordance with the Declaration of Helsinki.

From 2015 to 2020, a total of 182 patients, who underwent multidisciplinary presurgical evaluation for the treatment of epilepsy at the Epilepsy Center of Beijing Tiantan Hospital and Beijing Fengtai Hospital, were retrospectively screened.^13^, ^14^ The patients were diagnosed with DRE, which was defined as persistent seizures despite the use of two or more antiepileptic medications at the maximum tolerated doses. However, the patients did not undergo surgical resection. The clinicians recommended several palliative surgical approaches, such as corpus callosotomy (CC), VNS, and medication adjustment. A total of 93 patients were excluded due to the following reasons: (1) missing or low‐quality images, (2) history of intracranial surgery, (3) incomplete clinical data, and (4) loss of follow‐up. Their clinical information was extracted from electronic medical records.^15^, ^16^ The prognosis of the patients was predicted based on the data in the last follow‐up, including their seizure frequency before VNS implantation (pre‐VNS), drugs, and neuropsychiatric status in both VNS‐ and drug‐treated groups. However, the response (seizure reduction >50%) and McHugh classification^17^ were only recorded in the VNS‐treated group.

For the VNS‐treated patients, during the 3‐month pre‐VNS and 1‐year follow‐up period after the VNS treatment, the usage of the antiepileptic drugs (AEDs) remained unchanged. All the patients underwent the adjustment of stimulation parameters through the identical programming protocol. Approximately 2 weeks after implantation, the VNS generator was turned on with the following initial settings: current amplitude of 0.2 mA, frequency of 30 Hz, a pulse width of 500 μs, signal‐on time of 30 s, and signal‐off time of 5 min. The adjustments were performed every 2 or 3 weeks until the stimulation reached 1.0 mA. Then, the adjustments were performed after 1 month for the first 4 months followed by 4‐month intervals. The output current was gradually raised by 0.2–0.3 mA at each follow‐up visit until the seizures were reduced by ≥50%, the patient no longer tolerated the side effects, or the current reached a maximum of 3.5 mA.

### 2.2 Magnetic resonance imaging acquisition and preprocessing

All the participants underwent the same neuroimaging protocols for presurgical evaluation. The high‐resolution three‐dimensional (3D) T_1_‐weighted magnetization prepared rapid gradient echo (T_1_w MPRAGE) sequence (repetition time = 2300 ms; echo time = 2.53 ms; flip angle = 12°; slice thickness = 1 mm; no gap; voxel size = 1 mm × 1 mm × 1 mm) was performed using a 3T Siemens Verio scanner.^18^, ^19^

**FIGURE 1:**
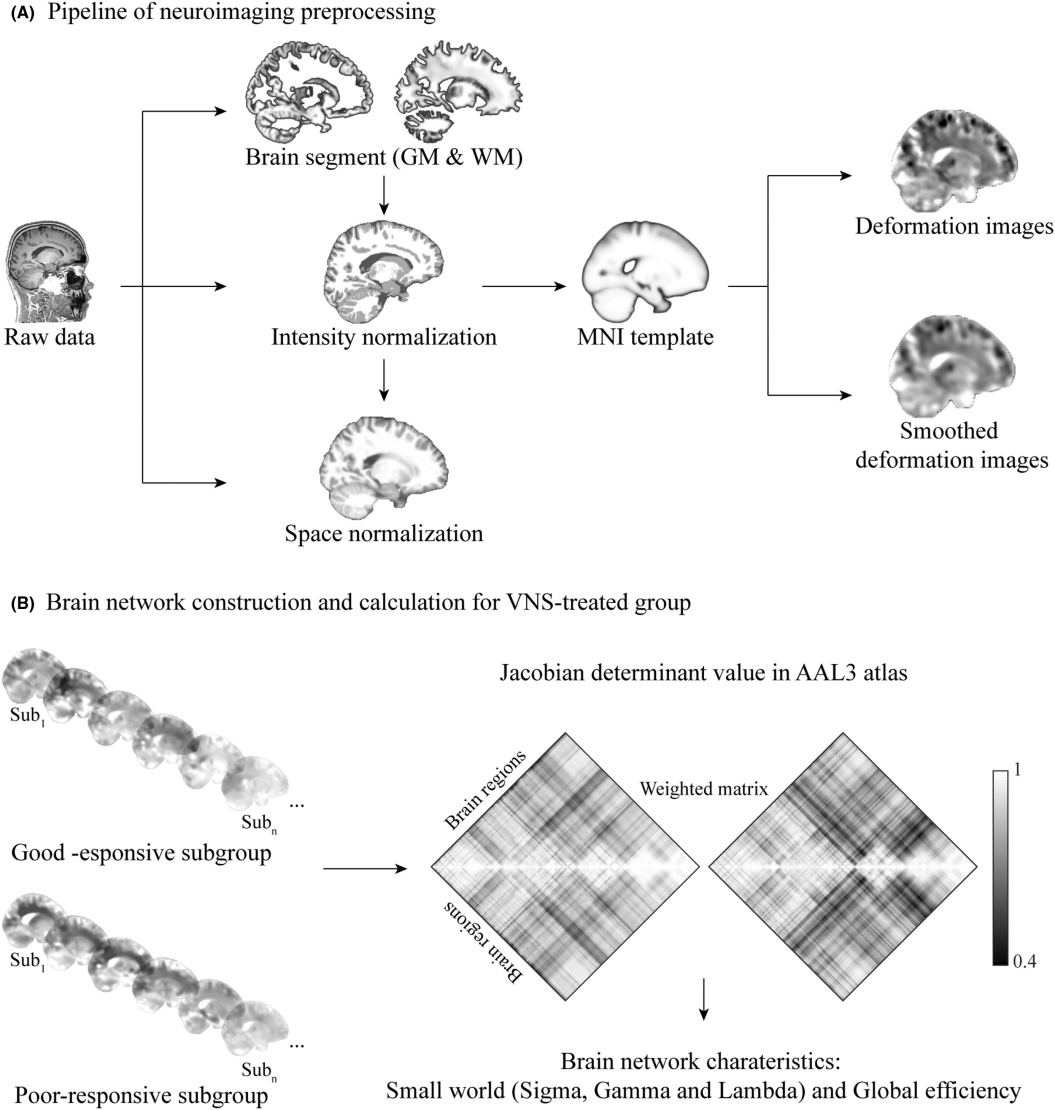
Brain network construction based on the structural deformation in images. (A) Overview of the image preprocessing procedures included the brain segment, intensity and space normalization, deformation calculation measured by Jacobian determinant, and smooth. (B) Regional average deformation between the VNS‐treated patients with good‐ and poor‐responsive outcomes was applied to construct the weighted matrix. The brain network characteristics, such as small worldness (sigma, gamma, and lambda) and global efficiency, were calculated and compared. GM, gray matter; WM, white matter; MNI, Montreal Neurological Institute; VNS, vagus nerve stimulation; AAL3, automated anatomical labeling 3.

The deformation in brain structure is a key component of brain signature. Therefore, in order to localize the differences in the brain structures between the groups, deformation‐based morphometry (DBM) was used to compare the differences in the relative volumes of brain structures using the Computational Anatomy Toolbox (CAT12: http://www.neuro.uni‐jena.de/cat/) for Statistical Parametric Mapping (SPM12, http://www.fil.ion.ucl.ac.uk/spm/). The preprocessing of the 3D T_1_w MRI scan was performed, followed by the preprocessing protocol, including intensity normalization, bias, and noise correction using the spatially adaptive nonlocal means (SANLM) filter, which removed the spatially varying noise while maintaining edges. The tissue segmentation divided the MRI scans into three classes, including gray matter (GM), white matter (WM), and cerebrospinal fluid (CSF). Next, segmentation was performed based on these three pure tissue classes as well as two additional mixed classes, including GM‐WM and GM‐CSF, using the partial volume estimation method for more precise segmentation. Then, the images were registered to a common reference template brain (IXI555 MNI152) in the Montreal Neurological Institute (MNI) stereotactic space using an affine and nonlinear (DARTEL and Geodesic Shooting) registration. In addition, the segmented images were modulated by scaling with the number of changes in the volume due to the nonlinear spatial registration, maintaining the total amount of GM and WM in the modulated image similar to that in the original image. A Jacobian matrix field was derived from the gradients of the deformation field and was used for the alignment of the individual brain to the previously created template. Then, the local Jacobian determinant of the deformation field, which was used to characterize the differences in the local volume, was written for each subject in normalized space. The Jacobian determinants were smoothed using an isotropic Gaussian smoothing kernel, expressed at its full width at half maximum of 5 mm. The smoothed Jacobian determinants were then used for further analysis^19^ (Figure 1A).

### 2.3 Brain network construction

The average Jacobian determinant values of the brain regions of each participant were extracted from the Automated Anatomical Labeling 3 (AAL3) atlas.^20^ The inter‐region correlations were calculated for the construction of the matrix. Then, the graph theoretical analysis was employed to investigate the network‐wide effects on the VNS patients with good or poor responses using the Brain Connectome Toolbox (BCT)^21^ and Graph Theoretical Network Analysis (GRETNA) toolbox,^22^ as described previously.^23^ The global topological characteristics, including small‐worldness (Sigma, Gamma, and Lambda characteristics) and global efficiency, were investigated and benchmarked to the weighted matrix. Each measurement was computed for a range of connection densities (5%–50%, with 5% increments) (Figure 1B).

### 2.4 Statistical analysis

The demographic data of the patients in the VNS‐ and drug‐treated groups were assessed using the Lilliefors test for normality. The homogeneity of variance was assessed using Levene's test. The categorical and continuous variables were compared and analyzed using the Pearson's χ^2^ or Fisher's exact test and Student's *t* test or nonparametric Mann–Whitney *U* test for the two dependent samples, respectively.

First, the correlations between brain abnormalities and epilepsy severity were analyzed as well as between the differences in the brain function status of the patients with different VNS responses using a regression model. Second, the brain‐clinical signature was defined as the collected variables, including gender, age, disease duration, age of seizure onset, etiology, imaging representation, seizure type, and epilepsy type, which were evaluated according to the International League Against Epilepsy (ILAE) guidance,^24^ intelligence status, memory alteration, pre‐VNS ASM, pre‐VNS seizure frequency, and brain deformation. The potential of the brain‐clinical signature to predict the VNS response was then compressively evaluated. Different regression models, including partial least square regression (PLS), ordinal logistic regression, and binary logistic regression were used for the analysis of seizure reduction (continuous), McHugh classification (ordinal), and response or nor (binary), which were selected as the data types of VNS response. The statistical mediation analysis was then performed using the SPSS PROCESS macro^13^ to determine whether the identified correlations between the independent predictors and VNS response were a by‐product of the statistical mediation. In detail, the X predicted Y (X → Y) solely or in part because X predicted M, which in turn predicted Y (X → M → Y).

Statistical significance was set at 5%. The statistical analyses were performed using SPSS Statistics 26.0 software (SPSS, Inc.).

## 3 RESULTS

### 3.1 Participants

**FIGURE 2:**
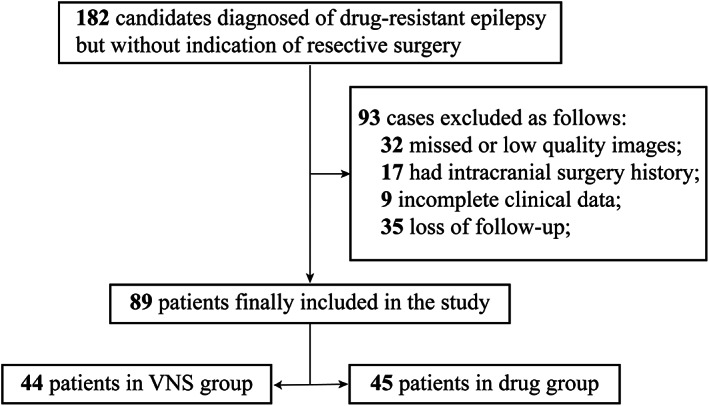
Flowchart of 182 consecutive candidates who were diagnosed with drug‐resistant epilepsy underwent presurgical evaluation and were not indicated for surgical resection. A total of 93 patients were excluded based on exclusion criteria. Finally, this study consisted of 89 patients, with 44 patients with VNS treatment and 45 with drug treatment.

**FIGURE 3:**
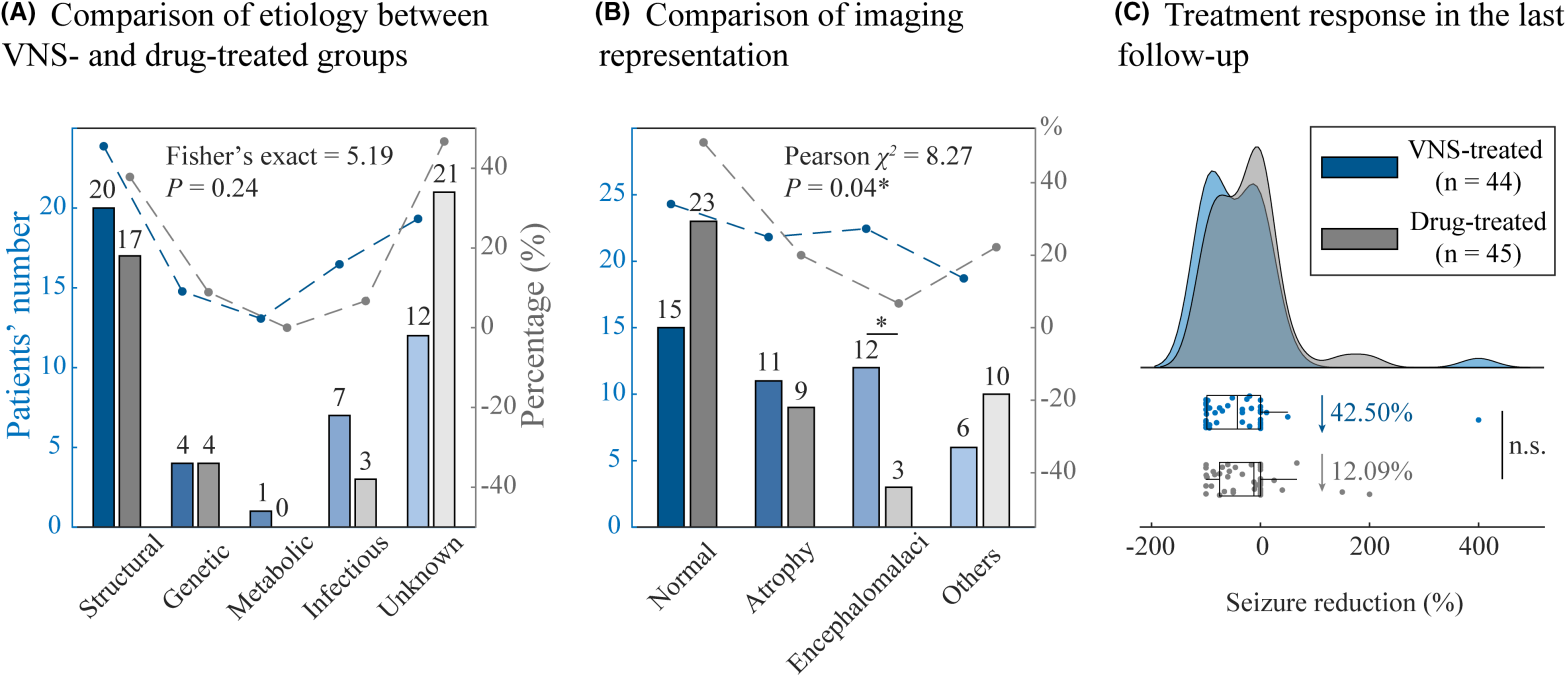
Determination of clinical variables affects the choice of VNS‐ or drug‐treated groups in the presurgical evaluation. (A) Overview of the etiology distribution between the two groups, showing no significant differences. (B) Overview of the imaging representation between the two groups. The patients with encephalomalacia were likely to have VNS implantation. The bar represents the patients' numbers and the dotted line represents the percentage in each cohort. *Significant difference. (C) Overview of the treatment response between the two groups. The efficacy of VNS was better than the drug treatment in the last follow‐up, showing no significant difference.

A total of 89 patients, who were diagnosed with epilepsy but did not undergo surgical resection, were finally included in this study. The patients were divided into the VNS‐treated group (*n* = 44) and the drug‐treated group (*n* = 45) according to the patients' or their guardians' preferences. The VNS‐treated group patients were further divided into the VNS good‐responsive and poor‐responsive subgroups based on the seizure reduction rate (> or <50%, respectively) in the last follow‐up (Figure 2). The demographic information of the patients, including their gender, age, disease duration, onset time of seizure, etiology, seizure type, epilepsy type, intelligence status, memory alteration, antiseizure medication, and seizure frequency, did not show significant differences between the VNS‐ and drug‐treated groups. However, as compared to the drug‐treated group, the imaging techniques showed more abnormalities in the VNS‐treated group (Pearson's χ^2^ = 8.27, *p* = 0.04) due to encephalomalacia (Pearson's χ^2^ = 7.07, *p*_corrected_ = 0.03) and longer follow‐up (Student's *t* = 3.79, *p* < 0.01) (Table 1). The statistical significance of the within‐group differences in the etiology, imaging representations, and treatment response in the last follow‐up of the patients in the VNS‐ and drug‐treated groups are presented in Figure 3.

**TABLE 1:** Demographic characteristics of patients

Variables	VNS‐treated group	Drug‐treated group	Statistic
Numbers	44	45	–
Gender (male, %)	17 (38.6%)	12 (26.7%)	Pearson's χ^2^ = 1.45, *p* = 0.23
Age (years)	14.83 ± 7.76	14.43 ± 9.67	Student's *t* = 0.21, *p* = 0.83
Disease duration (years)	8.44 ± 6.91	8.35 ± 7.57	Mann–Whitney *U* = 945.50, *p* = 0.72
Onset time of seizure (years)	6.39 ± 5.71	6.08 ± 5.85	Mann–Whitney *U* = 920.50, *p* = 0.57
Etiology	Structural (20, 45.5%)	Structural (17, 37.8%)	Fisher's exact *t* = 5.19, *p* = 0.24
	Genetic (4, 9.1%)	Genetic (4, 8.9%)	
	Metabolic (1, 2.3%)	Metabolic (0, 0%)	
	Infectious (7, 15.9%)	Infectious (3, 6.7%)	
	Unknown (12, 27.3%)	Unknown (21, 46.7%)	
Imaging representation	Normal (15, 34.1%)	Normal (23, 51.1%)	Pearson's χ^2^ = 8.27, *p* = 0.04*; differences from encephalomalacia (Pearson's χ^2^ = 7.07, *p*_corrected_ = 0.03*)
	Atrophy (11, 25.0%)	Atrophy (9, 20.0%)	
	Encephalomalacia (12, 27.3%)	Encephalomalacia (3, 6.7%)	
	Others (6, 13.6%)	Others (10, 22.2%)	
Seizure type	Focal (13, 29.5%)	Focal (13, 28.9%)	Pearson's χ^2^ = 3.57, *p* = 0.18
	Generalized (12, 27.3%)	Generalized (20, 44.4%)	
	Unknown (19, 43.2%)	Unknown (12, 26.7%)	
Epilepsy type	Focal (9, 20.5%)	Focal (9, 20.0%)	Likelihood ratio = 0.64, *p* = 0.92
	Generalized (17, 38.6%)	Generalized (19, 42.2%)	
	Combined (5, 11.4%)	Combined (3, 6.7%)	
	Unknown (13, 29.5%)	Unknown (14, 31.1%)	
Intelligence status	Normal (15, 34.1%)	Normal (10, 22.2%)	Pearson's χ^2^ = 1.55, *p* = 0.25
	Decline (29, 65.9%)	Decline (35, 77.8%)	
Memory alteration	Normal (14, 31.8%)	Normal (7, 15.6%)	Pearson's χ^2^ = 3.26, *p* = 0.09
	Decline (30, 68.2%)	Decline (38, 84.5%)	
Pretreatment ASM (*n*)	3.0 (2.0, 4.0)	2.0 (2.0, 3.0)	Mann–Whitney *U* = 868.50, *p* = 0.30
Posttreatment ASM (*n*)	3.0 (2.0, 3.0)	3.0 (2.0, 3.0)	Mann–Whitney *U* = 914.50, *p* = 0.52
Change of ASM (%)	0.0 (−12.5, 0.0)	0.0 (0.0,37.5)	Mann–Whitney *U* = 772.00, *p* = 0.05
Pretreatment seizure frequency (*n*)	60.0 (8.0, 150.0)	30.0 (4.0, 90.0)	Mann–Whitney *U* = 813.00, *p* = 0.15
Posttreatment seizure frequency (*n*)	8.5 (1.0, 90.1)	4.5 (1.5, 71.3)	Mann–Whitney *U* = 974.50, *p* = 0.90
Change of seizure frequency (%)	−42.5 (−98.2, 0.0)	−12.1 (−75.0, 0.0)	Mann–Whitney *U* = 799.50, *p* = 0.11
Response	22 (50.0%)	19 (42.2%)	Pearson's χ^2^ = 0.54, *p* = 0.53
McHugh classification	I (18, 40.9%)	–	–
	II (4, 9.1%)		
	III (7, 15.9%)		
	IV (0, 0%)		
	V (15, 34.1%)		
Follow‐up (months)	57.4 ± 17.5	42.2 ± 20.2	Student's *t* = 3.79, *p* < 0.01*

*Note*: Continuous data with normal distribution are represented as mean ± SD. The other data are presented as median (25th and 75th percentiles). Categorical data were represented as *n* (%).

Abbreviations: ASM, antiseizure medication; VNS, vagus nerve stimulation.

* *p* < 0.05.

### 3.2 Clinical variables and brain network characteristics for the prediction of VNS response

**FIGURE 4:**
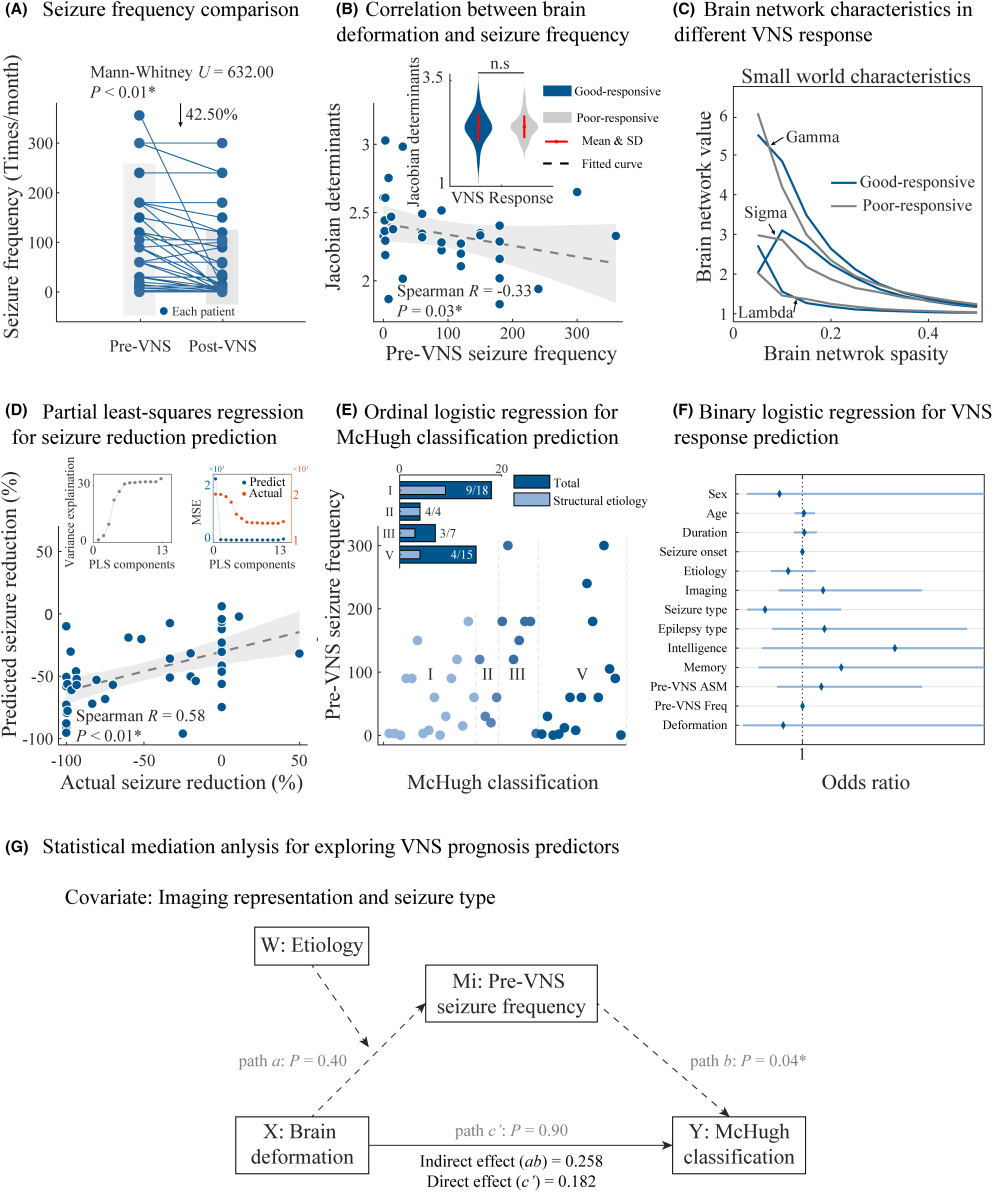
Brain‐clinical signature associated with the clinical phenotype and VNS response. (A) Seizure frequency significantly decreased by 42.50% after the VNS treatment in the entire cohort. (B) Pre‐VNS seizure frequency was significantly negatively correlated with the brain deformation measured using Jacobian determinants. There was no significant difference in brain deformation between VNS good‐ and poor‐responsive subgroups. (C) Altered small‐worldness characteristics along with the sparsity of the brain network. (D–F) Application of PLS, ordinal logistic regression, and binary logistic regression models to predict the VNS response based on the brain‐clinical signature (13 components). (G) Separate statistical mediation analysis identified the correlation between the brain deformation and McHugh classification (path *c*) but was only mediated by the pre‐VNS seizure frequency (path *b*). The brain deformation did not mediate the pre‐VNS seizure frequency (path *a*) and McHugh classification (path *c*′). *Statistical significance.

In this study, the patients experienced a significant improvement in their disease conditions after VNS treatment (Mann–Whitney *U* = 632.00, *p* < 0.01) with a decrease of 42.50% in the average seizure reduction (Figure 4A). The brain deformation did not show significant differences between the VNS good‐responsive and poor‐responsive subgroups (Student's *t* = 0.15, *p* = 0.88) (Figure 4B), normal and declined intelligence subgroups (Student's *t* = 0.17, *p* = 0.10), and normal and declined memory subgroups (Student's *t* = −0.36, *p* = 0.72) (Figure S1A).

In terms of brain signature, the brain deformation decreased significantly with the increase in the pre‐VNS seizure frequency (Spearman's *R* = −0.33, *p* = 0.03) (Figure 4B). Furthermore, the brain‐network characteristics of the VNS good‐responsive subgroup, including small worldness and global efficiency, were higher as compared to those of the VNS poor‐responsive subgroup across the sparsity of >0.10 (Figure 4C). The global efficiency of the brain network did not show significant differences between the VNS good‐responsive and poor‐responsive subgroups (Student's *t* = 0.13, *p* = 0.90) (Figure S1B).

For the prognosis prediction of VNS treatment, the clinical and brain signatures were analyzed based on three different prognostic evaluation parameters, including seizure reduction, McHugh classification, and VNS response. The PLS regression model illustrated that the brain‐clinical signatures could successfully predict seizure reduction, which was indicated by the model in achieving the best variance explanation in all the included 13 components. The actual seizure reduction (response values) was significantly correlated with the predicted seizure reduction (predicted values) (Spearman's *R* = 0.58, *p* < 0.01) with the low mean‐square error of the residuals (−3.34%) derived from the regression model (Figure 4D). Meanwhile, the ordinal logistic regression model was a good fit (χ^2^ = 295.37, *p* < 0.01). The variable pre‐VNS seizure frequency and structural etiology showed a statistically significant effect on the McHugh classification. A single unit increase in the pre‐VNS seizure frequency could increase the log of odds of the higher McHugh classification (worse prognosis) by 4.99 ([estimate = 0.01, χ^2^ = 4.99, *p* = 0.03, 95% confidence interval (95% CI) = −0.012 to −0.001]). In contrast to the genetic, metabolic, infectious, and unknown etiologies, the structural etiology was correlated with a lower McHugh classification (better prognosis) (estimate = −2.17, χ^2^ = 3.90, *p* < 0.05, 95% CI = −4.322 to −0.017) (Figure 4E). The binary logistic regression model was a good fit (χ^2^ = 5.77 and *p* = 0.48 in the Hosmer and Lemeshow test, *p* = 0.48 in the Omnibus test, and Nagelkerke *R* ^2^ = 0.48). All the brain deformation and clinical variables (average whole‐brain Jacobian determinant values) did not show a significant correlation with the VNS response (Figure 4F).

The effects of brain alterations on the disease severity and prediction of VNS treatment prognosis were further explored. The statistical mediation analysis, controlling the imaging representation and seizure type, showed that there was no mediation, suggesting that the correlation between the pre‐VNS seizure frequency and McHugh classification was not a by‐product of the increase in the brain deformation (path *a*: *R* = 0.35, *p* = 0.40; path *c*′: effect = 0.18, *p* = 0.90, boot standard error [SE] = 1.49, 95% CI = −2.83 to 3.19). However, the McHugh classification was mediated by the pre‐VNS seizure frequency (path *b*: *R* = 0.47, *p* = 0.04) (Figure 4G).

## 4 DISCUSSION

The appropriate selection of candidates and accurate prediction of VNS response are crucial for the clinical treatment of epileptic patients. This selection and prediction can maximize the treatment effects of neuromodulation on DRE patients without indicating surgical resection. First, this study retrospectively showed a 42.5% seizure reduction after VNS treatment in the study cohort. Then, in order to comprehensively predict the VNS treatment response, brain bioinformatics was innovatively utilized and the clinical variables were analyzed. Finally, the brain‐clinical signatures were predicted, which could potentially interpret the clinical representations of DRE patients, who underwent VNS.

### 4.1 Efficacy of VNS was better than the drug treatment

VNS has been widely used as an adjuvant therapy among DRE patients, who are not eligible for surgical resection. The US Food and Drug Administration (FDA) approved VNS for use in adult and young epileptic patients over the age of 12 in 1997. Numerous studies have previously reported the efficacy and safety of VNS treatment for DRE patients. A meta‐analysis analyzed 74 clinical studies, including 3321 patients, and reported that the seizure frequency decreased by an average of 36% (at 3–12 months after surgery) to 51% (after more than 1 year of surgery).^25^ In the present study cohort, the seizure reduction among the patients, who underwent VNS treatment, was 42.5%, which was better than that of the drug treatment of 12.09%; these results were consistent with those of the previous study mentioned earlier. Another recent meta‐analysis, which analyzed 778 studies, including 2869 patients, suggested that the response rate might increase from 49% at 0 to 4 months after VNS treatment to 63% at 24 to 48 months.^11^ These results suggested the VNS treatment could allow progressive and time‐dependent seizure control.^26^ This phenomenon could benefit from the emerging evidence of neuromodulation programming. A recent study observed the VNS response at 1.625 mA, 30 s ON, 3 min OFF.^27^ The current study showed that more programming experience could improve VNS treatment. In terms of the response rate, a seizure reduction of >50% was observed in 49.6% of the patients,^25^ which was consistent with the results of the present study (50% of the patients showed similar treatment effects). In addition, the meta‐analysis also reported that the mean decrease in seizure frequency after VNS treatment was greater in patients with generalized epilepsy (57.5%), epileptic type of tuberous sclerosis (68.1%), and trauma (78.6%).^25^

### 4.2 Clinical factors could affect the treatment strategy of VNS or drug adjustment

The DRE patients, who do not undergo surgical resection, usually face the dilemma of treatment choice. Based on our experience, the VNS treatment was preferred for the DRE patients with exact etiology over the drug adjustment (32 vs. 24). However, it was identified that we were more conservative to adjust the dosage or type of medication rather than VNS implantation (21 vs. 12) to avoid the anesthesia and surgical risks for the patients with unknown etiology. There was a similar trend in the patients' imaging representations. The VNS treatment was preferred over drug treatment for the patients who displayed obvious brain abnormalities, such as widespread atrophy or encephalomalacia, resulting from encephalitis or trauma. However, for the DRE patients with normal MRI, drug adjustment was selected until more information was obtained for deciding on further treatment.

### 4.3 Brain‐clinical signature for VNS response

An important feature of this study was the innovative introduction of brain characteristics. In the past, the common structural alterations, such as the cortical thickness and surface area, in the brain of patients treated with VNS could not be identified using the traditional quantitative computational approaches at the whole‐brain level. Therefore, a published fMRI study only focused on the thalamocortical connectivity^28^ and several patients presented with encephalitis or previous surgical cavity showed heterogeneity. Another study utilized the connectome profile for the identification of VNS response but did not show the MRI representation of the included patients. The variability in white matter microstructure might affect the statistical results. On the other hand, all the included patients with normal MRI presentations might also have some bias.^29^ Furthermore, other studies used electromagnetic signal‐related methods, such as resting‐state magnetoencephalography‐based network topology^30^ and electroencephalography symmetry^31^ for the prediction of VNS prognosis. However, these attempts could not identify the brain alterations or indicate etiology. Considering these challenges, in this study, the brain deformations were calculated by quantitatively measuring the brain alterations to explore their correlation with seizure severity and VNS prognosis. Generally, the decrease in structural deformation was correlated with the increase in seizure attacks. It was supposed that a mild or subtle cortical dysgenesis might exist, resulting in severe seizures, but could not be identified in the current situation. However, the epileptogenicity of trauma and encephalitis‐related lesions was weaker than that of cortical dysgenesis. The brain functional status was also investigated and was similar in the patients with good and poor VNS responses. However, the patient with good VNS response had a relatively healthier and more efficient brain function, indicating their ability to coordinate physiological function and neutralize the negative effects of epilepsy.

Currently, there are different approaches, including the continuous rate of seizure reduction, ordinal measurement of McHugh classification, and binary measurement of response, for evaluating the efficacy of VNS treatment. This study analyzed the comprehensive correlations of clinical and brain variables with the treatment outcomes. First, the study concluded that the brain‐clinical signature had the moderate potential to predict seizure reduction. These results should be further validated in future studies and should be extended by other potentially useful variables.

**FIGURE 5:**
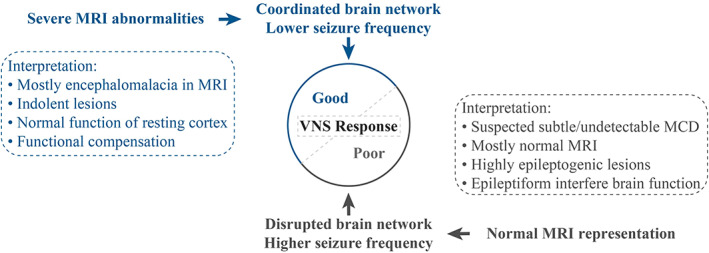
Summary of the findings and interpretation of MRI representation, brain function status/epileptogenic network, seizure severity, and VNS response outcomes. MRI, magnetic resonance imaging; VNS, vagus nerve stimulation; MCD, malformation of cortical dysplasia.

The current study further showed that the pre‐VNS seizure severity and mild MRI abnormalities were positively correlated with the worse prognosis. The potential of MRI representation for VNS response prediction is controversial. Some researchers considered that there was no correlation between the presence or absence of lesions in the MRI representation and the VNS responsiveness.^32^, ^33^ Other researchers showed that the absence of a lesion in the MRI representation was significantly correlated with a better seizure outcome following VNS.^11^, ^34^, ^35^ However, other studies showed contradictory results, showing that the presence of brain lesions in the MRI representation could predict the freedom from a seizure after VNS.^36^ The results in this study were consistent with the latter study and could be explained as follows. Severe MRI abnormalities mainly resulted from the neonatal intracranial hemorrhage, while these pathological alterations were indolent, and the resting normal cortex might compensate for the brain function and maintain coordination. Therefore, these patients might be amendable to VNS. Conversely, the patient with normal brain images could not rule out the possibility of having mild or subtle epileptogenic lesions, which could not be detected using the current visual analysis techniques. Therefore, the highly epileptogenic lesions might disrupt normal brain function while dominating the epileptic network. These patients suffered from more severe seizure attacks and benefitted less from the palliative treatment (Figure 5). The conclusions of Arya et al were contradictory to those of this study. However, in their study, the main difference was observed in the MRI diagnosis; most of the patients were diagnosed with highly epileptogenic pathology, such as malformation of cortical dysplasia, mesial temporal sclerosis, and tuberous sclerosis complex.^35^ On the other hand, in the current study, most of the patients were diagnosed with indolent lesions, such as brain injury or intracranial hemorrhage.

The statistical analysis could not identify any significant variable, which could predict the outcomes of binary VNS response. It was previously reported that seizure freedom could be significantly predicted by the age of epilepsy onset >12 years.^11^ This result supported the result of the current study, which showed that highly epileptogenic lesions, such as focal cortical dysplasia, preferably occurred at a young age. It was also reported that a short epilepsy duration was likely to be associated with a better prognosis,^35^ which could be explained that the brain network was not largely affected by the epileptic activity and was restored by the VNS treatment. The statistical median analysis suggested that the VNS prognosis, evaluated by McHugh classification, could be predicted by the pre‐VNS seizure severity, thereby further validating the hypothesis. However, the prognosis could not be predicted by brain deformation, suggesting that the brain signature should take other measurements, such as dynamic status calculation and brain structural quantification, into consideration.

### 4.4 Limitations

There were certain limitations to this study. First, this retrospective study was limited by the treatment choice of each patient. Hence, a randomized, prospective design is required to validate these results. Second, heterogeneous MRI representations were common in VNS patients. Despite considering the Jacobian determinants as the quantitative features for analysis, more potentially useful features might have been underestimated, which should be discovered in future studies. Third, although the included patients were selected from a relatively larger sample of patients (*n* = 182), the sample size of the study was small.

## 5 CONCLUSIONS

VNS is a common procedure, which has been widely applied in the clinical treatment of epilepsy. The consistent identification of good candidates for VNS treatment using presurgical evaluation parameters is challenging because some patients might benefit from surgery, while others might not. To date, no single clinical factor has been reproducibly determined as a predictor of VNS prognosis. This study presented a brain‐clinical signature, which was associated with the clinical phenotype and VNS responsiveness, which might allow clinicians to personalize the treatment decisions for DRE patients.

## AUTHOR CONTRIBUTIONS

Zhihao Guo and Jiajie Mo contributed to the study methodology, software, formal analysis, and writing – original draft. Chao Zhang contributed to the study methodology and formal analysis. Jianguo Zhang contributed to the study conceptualization and visualization. Wenhan Hu obtained resources, carried out data curation, project administration, and writing – review and editing. Kai Zhang supervised, carried out project administration, writing – review and editing, and acquired funding.

## FUNDING INFORMATION

This work was supported by the National Key R&D Program of China (2021YFC2401201), Capital's Funds for Health Improvement and Research (2022‐1‐1071, 2020‐2‐1076), and the National Natural Science Foundation of China (82071457, 82271495, 82201603, 82201600).

## CONFLICT OF INTEREST

None of the authors has any conflict of interest to disclose. We confirm that we have read the Journal's position on issues involved in ethical publication and affirm that this report is consistent with those guidelines.

## CONSENT TO PARTICIPATE

All the patients provided informed consent for the use of their medical records.

## Supporting information


Figure S1

## Data Availability

The data sets generated and analyzed during the current study are available from the corresponding author upon reasonable request.
