# Supplementary figures and images for: Brain‐clinical signatures for vagus nerve stimulation response
Source: CNS Neurosci Ther. 2022 Nov 22;29(3):855–65. doi: 10.1111/cns.14021 (PMC9928539; doi:10.1111/cns.14021)

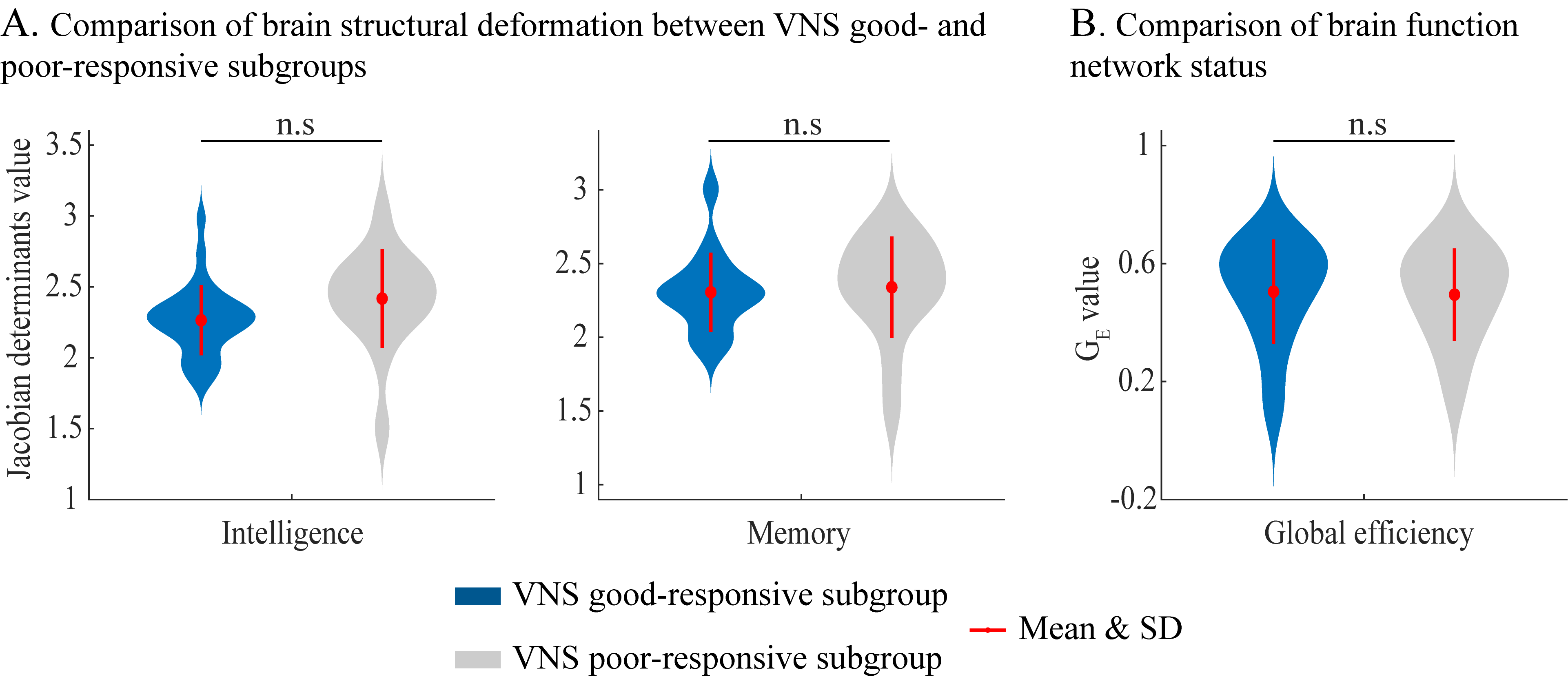

Supplement: Supplementary file 1 — Figure S1 [file CNS-29-855-s001.tif]
